# Advances and Limitations of Antibody Drug Conjugates for Cancer

**DOI:** 10.3390/biomedicines9080872

**Published:** 2021-07-23

**Authors:** Candice Maria Mckertish, Veysel Kayser

**Affiliations:** Sydney School of Pharmacy, Faculty of Medicine and Health, The University of Sydney, Sydney, NSW 2006, Australia; cmck9835@uni.sydney.edu.au

**Keywords:** antibody drug conjugates, drug resistance, linkers, payloads, therapeutic index, target specific, ADC clearance, protein aggregation

## Abstract

The popularity of antibody drug conjugates (ADCs) has increased in recent years, mainly due to their unrivalled efficacy and specificity over chemotherapy agents. The success of the ADC is partly based on the stability and successful cleavage of selective linkers for the delivery of the payload. The current research focuses on overcoming intrinsic shortcomings that impact the successful development of ADCs. This review summarizes marketed and recently approved ADCs, compares the features of various linker designs and payloads commonly used for ADC conjugation, and outlines cancer specific ADCs that are currently in late-stage clinical trials for the treatment of cancer. In addition, it addresses the issues surrounding drug resistance and strategies to overcome resistance, the impact of a narrow therapeutic index on treatment outcomes, the impact of drug–antibody ratio (DAR) and hydrophobicity on ADC clearance and protein aggregation.

## 1. Introduction

Conventional cancer therapy often entails a low therapeutic window and non-specificity of chemotherapeutic agents that consequently affects normal cells with high mitotic rates and provokes an array of adverse effects, and in some cases leads to drug resistance [1]. Monoclonal antibodies (mAbs) have evidently demonstrated great therapeutical potential for the treatment of a multitude of ailments, particularly for cancer [2]. Prior to the development of ADCs, mAbs attracted interest attributable to its target specificity, wide therapeutic index, and its affiliation with fewer side effects, in particular for cancer [3], compared to conventional therapy, which incorporates chemotherapy, radiation therapy and surgery [4]. As an improvement, ADCs provide a synergistic effect upon the conjugation of a mAb to a cytotoxic drug, compared to the mAb used alone [4,5].

**Figure 1:**
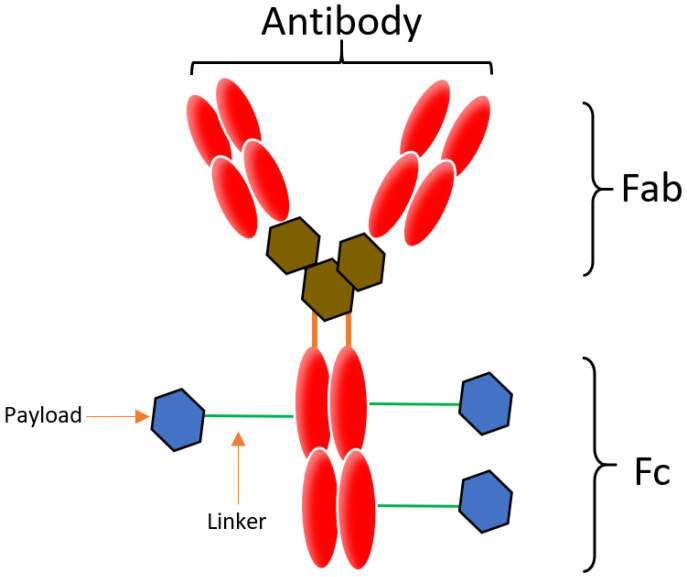
Basic structure of an ADC: An ADC is comprised of the monoclonal antibody (red), linker (green) and payload. The payload can be conjugated to different parts of the mAb and is commonly attached via cysteine (brown) or lysine residues (blue). Generally, more than one payload can be attached. An IgG antibody structure consists of two Fab fragments. Each fragment consists of one heavy chain and one light chain and the Fc fragment.

Conjugation is an approach that enables the attachment of highly toxic drugs to a tumor specific mAb chemically, in order to construct an ADC. An ADC is comprised of a mAb, a linker and a cytotoxic payload [1,6], illustrated in Figure 1. The linker conjugates the payload to the mAb, which binds to the target that is over expressed on the tumor cell, and the payload potentiates the therapeutic action [1]. ADC payloads should exert stability in storage and in the blood stream as well as have non-immunogenic effects. The main characteristics of ADC include a good internalization rate, low immunogenicity, high binding specificity and affinity, a potent payload, and a stable linker [7].

Amongst emerging antibody-based therapies, ADCs have demonstrated superior effects over standard chemotherapeutics for cancer [8]. Amidst the growing enthusiasm for ADCs, their design and development entail many challenges, including susceptibility to degradation due to the required low storage temperature and anomalies that arise during transport. Moreover, although high formulation concentrations, through SC (subcutaneous) or IV (intravenous), attracted interest, this often leads to lack of stability or high aggregation propensity in formulation. Alternatively, low concentration reduces bioavailability and ability to penetrate the tissue to exert effects. The challenge lies in obtaining the optimal concentration in formulation to enhance local bioavailability and tissue penetration [9,10]. Currently, over 100 ADCs are undergoing clinical trials, out of which approximately 20% of ADCs have been terminated or withdrawn during either phase I or phase II, a few ADCs were linked to dose limiting toxicities [11]. A narrow therapeutic index results in toxicity [12]. Attaining an optimal dose for an ADC is important in limiting the dosing cycles tolerated by the patient, dose reductions, skipped doses or discontinuation in the therapy. There have been clinical and translation dosing schedules, including a fractionated dosing schedule, to address dose limiting toxicities in patients [13,14,15,16,17]. For example, Mylotarg^®^ caused a high rate of fatal toxicities related to the dosing schedule and failed to display any amelioration in efficacy compared to standard chemotherapy and hence was withdrawn from the market in 2010 [4,18]. Over recent years ADC research has entailed the optimization of current ADCs, and the design and development of various linkers that are delineated in the following sections.

## 2. Overview of ADCs

The interest in ADCs has increased remarkably in recent years. As mentioned, more than 100 ADCs are currently undergoing clinical trials for cancer indications. The majority of ADCs have progressed from phase I to phase II. Phase III trials have shown promising results for some ADCs; Mirvetuximab soravtansine (NCT02631876), indicated for the treatment of ovarian cancer, and [vic]-trastuzumab duocarmazine (NCT03262935), indicated for the treatment of breast cancer, are amongst a few that have progressed to phase III trials. Some of the ADCs in clinical trials have employed cleavable linkers such as Valine-Citrulline (Val-Cit) due to its stability in circulation. Most of the ADCs are CD targeting, except for Tusamitamab ravtansine (SAR408701) which targets carcinoembryonic antigen-related cell adhesion molecule 5 (CEACAMS) indicated for non-small cell lung cancer (Phase III NCT04154956) [11,19].

As of 2021, there has been nine ADCs that have successively been approved. However, toxicity profiles have been reported, e.g., the toxicity reports on Mylotarg^®^ mentioned previously, notwithstanding the indicated clinical benefits [20]. Other previously approved ADCs include Brentuximab vedotin (Adcetris^®^: Seattle genetics), Inotuzumab ozogamicin (Besponsa^®^: Pfizer), both approved for hematological malignancies, additionally, trastuzumab emtansine (Kadcyla^®^: Genentech) approved for breast cancer. These ADCs target the CD30 receptor, CD22 receptor and HER2 (human epidermal growth factor receptor 2) receptor, respectively [1,14]. Five other ADCs have been added between 2019–2021 to the list of approved ADCs, including Polatuzumab vedotin (Polivy^®^: Genentech/Roche) targets CD79b indicated for relapsed or refractory diffuse large B-cell lymphoma, Sacituzumab govitecan (IMMU-132) (Trodelvy^®^: Gilead Sciences) targets TROP-2 for the treatment of triple negative breast cancer, [fam]-trastuzumab deruxtecan (Enhertu^®^: Daiichi Sankyo/AstraZeneca) indicated for HER2+ metastatic breast cancer and targets HER2, Enfortumab vedotin (Padcev^®^: Astellas Pharma/Seattle Genetics) targets nectin 4 for the treatment of urothelial cancer, Belantamab mafodotin (Blenrep^®^: GlaxoSmithKline) targets BMCA and is indicated for multiple myeloma.

### 2.1. Gemtuzumab Ozogamicin

Gemtuzumab ozogamicin (Mylotarg^®^: Pfizer/Wyeth) was the first ADC that was developed. It was an anti-CD33 humanized antibody indicated for relapsed acute myeloid leukemia in 2000. It was withdrawn from the market in 2010 as a result of safety concerns and a high rate of fatal toxicities. However, Mylotarg^®^ was reapproved in 2017 and 2018 by the FDA (Food and Drug Administration) and the EMA (European Medicines Agency), respectively, for newly diagnosed acute myeloid leukemia (AML) and relapsed/refractory acute myeloid leukemia [18,20]. It was approved after a fractionated dosing schedule (previously administered as one dose of 9 mg/m^2^ that was altered to 3 doses of 3 mg/m^2^) was introduced which decreased the maximum plasma concentration and enhanced the safety profile and response rate [1,14].

Its reapproval was based on a lower dose recommendation, an altered dosing schedule and a different patient population. It is used in combination with chemotherapy or as a stand-alone treatment. It is conjugated via an acid cleavable hydrazone linker, 4-(4-acetylphenoxy) butanoic acid (AcBut) to a cytotoxic agent (N-acetyl gamma calicheamicin) [21,22]. It has an average DAR of 2 to 3 [23]. The linker moiety affixes to exposed lysines (lys) on the antibody. Attachment to the target receptor CD33 is followed by internalization of the ADC and, subsequently, release of the cytotoxic agent into the acidic environment by hydrolysis of the hydrazone in the lysosomes [24]. Calicheamicin provokes its cytotoxic effect by binding to deoxyribonucleic acid (DNA) ensuing breakage in the DNA-double strand and thereby cell death [20,25]. The clearance rate is 0.35 L/h and the half-life after the first dose is 62 h, and 90 h after the second dose [24].

### 2.2. Brentuximab Vedotin

Brentuximab vedotin (Adcetris^®^: Seattle genetics) targets CD30 on the surface of cancer cells. The antibody is linked to the payload, monomethyl auristatin E (MMAE), via a protease cleavable linker Val-Cit. The linker is conjugated to cysteine (cys) residues on the antibody. It has a DAR of 4. Brentuximab vedotin, upon attaching to CD30, is internalized and then transported to lysosomes. Cleavage by proteases in the lysosome releases MMAE into the cytoplasm to exert it cytotoxic effects by inhibiting microtubule polymerization inducing apoptosis via cell cycle arrest [26]. Brentuximab was approved in 2011 by the FDA for the treatment of anaplastic large cell lymphoma (ALCL). In 2018, the FDA approved the coadministration of Brentuximab vedotin with chemotherapy (vinblastine, doxorubicin and dacarbazine) for treating adult patients who had previously untreated stage III or IV classical Hodgkin lymphoma (HL). The EMA has authorized conditional approval for brentuximab vedotin. In terms of its metabolic profile, the ADC has a half-life of 4–6 days and an estimated clearance of 1.457 L/day [27].

### 2.3. Inotuzumab Ozogamicin

Inotuzumab ozogamicin (Besponsa^®^: Pfizer/Wyeth) targets CD22. The payload N-acetyl-gamma-calicheamicin is covalently attached to the linker 4-(4-acetylphenoxy) butanoic acid (ActBut) which is bound to exposed lysine residues. It has a DAR of 6 [28]. Inotuzumab ozogamicin has a high affinity for the target antigen CD22 on the cancer cell [29]. After binding to the target antigen, the ADC is then internalized into the lysosomal compartment. The payload is released and then exerts its effects, resulting in the double strand cleavage of DNA leading to apoptosis and cell cycle arrest. It was approved in 2017 by the FDA and EMA. It is used as a monotherapy for the treatment of relapsed/refractor B-cell acute lymphoblastic leukemia (ALL). The ADC has a half-life of 12.3 days and a clearance of 0.0333 L/h [30].

### 2.4. Trastuzumab Emtansine

Trastuzumab emtansine (Kadcyla^®^: Genentech/Roche) was approved by the FDA and EMA in 2013 for the treatment of HER2+ metastatic breast cancer. The cytotoxic payload mertansine (DM1) is attached to the antibody via a non-reducible thioether linker 4-(N-maleimidomethyl) cyclohexane-1-carboxylate (MCC) to lysine residues on trastuzumab [31]. The ADC binds to the target receptor HER2, then enters the cell via receptor mediated endocytosis leading to the proteolytic degradation of only the antibody portion of trastuzumab emtansine in the lysosomes. The lysine-MCCDM1 portion of the antibody is released into the cytosol where it binds to microtubules inducing apoptosis and cell cycle arrest. The DAR of DM1 is 3.5. In terms of the metabolic profile of T-DM1, it has a half-life of 4 days and a clearance of 0.68 L/day [31].

### 2.5. Polatuzumab Vedotin

Polatuzumab vedotin (Polivy^®^: Genentech/Roche) was approved by the FDA in 2019 and by the EMA in 2020. It is used in combination with bendamustine and rituximab for the treatment of relapsed or refractory diffuse large B-cell lymphoma. The ADC is conjugated to MMAE via engineered cysteines (THIOMABs) via a cleavable dipeptide linker, Valine-Citrulline (Val-Cit). It has a DAR of 3.5. It targets CD79b, which is expressed on the surface of B-cells [19]. The ADC binds to the target antigen. It is then internalized and undergoes proteolytic cleavage. MMAE is then released into the cytosol and binds to tubulin and inhibits it polymerization resulting in G2/M phase arrest and apoptosis [32]. The half-life of the ADC is 12 days with an estimated clearance rate of 0.9 L/day [33].

### 2.6. Sacituzumab Govitecan

Sacituzumab govitecan (IMMU-132) (Trodelvy^®^: Gilead Sciences/Immunomedics Inc.) was approved by the FDA in 2020. It received accelerated approval by the EMA in 2021 for triple negative breast cancer [19]. Triple negative breast cancer is defined by lack of expression of all three receptors generally found in breast cancer subtypes: the estrogen receptor (ER), progesterone receptor (PR) and human epidermal growth factor receptor 2 (HER2) [34]. It targets the tumor associated calcium signal transducer 2 (TROP-2) receptor which is generally expressed in high levels in triple negative breast cancer [35]. The payload, SN-38, is a topoisomerase I inhibitor that is linked to the antibody via a hydrolysable linker (pH sensitive linker), CL2A. It binds to the antibody at cysteine residues. It has a DAR of 7.6 [36]. The ADC binds to TROP-2 receptor and enters the cell, the payload SN-38 is released via hydrolysis of the linker and exerts it effects by preventing religation of topoisomerase I induced single strand breaks. The half-life of the ADC is 16 h and clearance is 0.002 L/h/kg [36].

### 2.7. [Fam]-Trastuzumab Deruxtecan

[fam]-trastuzumab deruxtecan (Enhertu^®^: Daiichi Sankyo/AstraZeneca) was approved in late December 2019 by the FDA and was provided conditional approval in 2021 by the EMA for the treatment of metastatic breast cancer and gastric cancer. The ADC is comprised of a topoisomerase I inhibitor payload, deruxtecan (DXd) via a cleavable tetrapeptide based linker. The linker is attached to cysteine residues on the antibody [19]. It binds to the HER2 on tumor cells, and is internalized, the linker is then cleaved by lysosomal enzymes. The payload is released and causes DNA damage leading to apoptosis. The ADC has an approximate DAR of 8 [37]. It was approved for the treatment of adult patients who have received two or more prior anti-HER2 based regimens such as trastuzumab, trastuzumab emtansine or pertuzumab for metastatic breast cancer. It is currently undergoing further clinical trials: DESTINY-Breast03 (NCT03529110) [38], to compare trastuzumab emtansine and [fam]-trastuzumab deruxtecan for unresectable and/or metastatic breast cancer previously treated with trastuzumab and taxane, and DESTINY-Breast02 (NCT03523585) [39], to observe the efficacy of [fam]-trastuzumab deruxtecan in pre-treated HER2 breast cancer that cannot be surgically removed or has spread. The half-life is 5.7–5.8 days, and the clearance is 0.42 L/day [37].

### 2.8. Enfortumab Vedotin

Enfortumab vedotin (Padcev^®^: Astellas Pharma/Seattle Genetics) was approved by the FDA in 2019 for locally advanced or metastatic urothelial cancer. It has not been approved by the EMA. The antibody is conjugated to the MMAE payload via a protease cleavable linker cleavable Maleimidocaproyl Valine-Citrulline (MC-Val-Cit) at cysteine residues on the antibody. It targets the cell surface protein nectin 4, a microtubule inhibitor [19,40]. It has a DAR of 3.8 [40]. Once the ADC is internalized, the linker is cleaved by proteases and the payload is released. MMAE then binds to tubulin and inhibits microtubule polymerization leading to apoptosis. The half-life is 3.4 days, and the clearance is 0.10 L/h [40].

### 2.9. Belantamab Mafodotin

Belantamab mafodotin (Blenrep^®^: GlaxoSmithKline) was approved by the FDA and EMA in 2020 for relapsed or refractory multiple myeloma. It is comprised of an afucosylated monoclonal antibody conjugated to microtubule disrupter monomethyl auristatin-F (MMAF) via a protease-resistant maleimidocaproyl (MC) linker which is attached to cysteine residues. It targets the B cell maturation antigen (BCMA) [19,41]. The antibody is afucosylated at the Fc region, this enhances binding to the Fc region and enhances antibody mediated cytotoxicity. Once the ADC binds to BCMA it is cleaved by proteases and releases the small molecule MMAF. MMAF then disrupts the microtubule polymerization leading to apoptosis. The DAR is approximately 4 [19,41]. The terminal half-life is 12 days after the first dose and 14 days at steady state. The clearance is 0.9 L/day after the first dose and 0.7 L/day at steady state [41]. 

Emerging new strategies addresses the issue encircling the therapeutic index. Some strategies involve the use of payloads with a lower potency profile. These strategies will be further elucidated in the following sections. Table 1 summarizes approved and marketed ADCs as of 2021, indicated for the treatment of different cancers. It also summarizes the DAR, clearance and half-life of the ADCs. Each ADC listed in the table has a unique target site, employing various linkers, cytotoxic payloads and conjugation strategies mentioned above.

## 3. Conjugation Chemistry of ADCs

Generally, antibody conjugation to cytotoxic agents commonly involves conjugation to exposed residues including lysines or reduction of disulfide bonds to expose free interchain cysteines on a therapeutic IgG (Immunoglobulin G) antibody. There are other, more recent approaches that introduce conjugation sites to the mAb such as site specific glycan conjugation, cysteine engineering, incorporation of unnatural amino acids and coupling short peptide tags to drug-linkers [12]. There are typically 80 lysine residues on an antibody; however, less than ten residues are chemically accessible for conjugation [42]. Cysteine conjugation eventuates in the reduction of four interchain disulfide bonds. These bonds are reduced under specific conditions and subsequently result in two, four, six or eight exposed sulfhydryl groups [4]. Both Cys and Lys conjugation methods result in heterogeneous mixtures [43]. Provided that mAbs already have an intrinsic heterogeneity due to glycosylation, this enhanced heterogeneity, in turn, makes it difficult to predict the efficacy, stability, pharmacokinetics, and therapeutic window of ADCs [43,44].

Modification of payloads with various functional groups, such as amine and thiol groups, to optimize conjugation as well as the impact of DAR, hydrophobicity on ADC clearance and improving conjugation methods to attain a homogenous ADC population, has been the primary focus of research in the field [45,46,47]. A high DAR and hydrophobicity entails the risk of immunogenic reactions and rapid clearance rates (further discussed in Section 5). Payload conjugation with native residues to produce a homogenous ADC population can be difficult. Apart from this, linker stability in the circulation is essential to avoid off target toxicities [48].

## 4. Linkers

As mentioned above, linkers are an integral component in maintaining the stability of the ADC in the systemic circulation and releasing the payload succeeding internalization at the target site. They have a pivotal role, critical for the stability and homogeneity of the ADC. An unstable linker can release the cytotoxic small molecule in the blood circulation before it reaches the target site, leading to undesirable systemic toxicities [49,50]. There are several classes of linkers including pH-dependent linkers, disulfide linkers and enzyme labile (peptide based) linkers. 

Linkers are subdivided in two categories, cleavable and non-cleavable, based on their release mechanism. Table 2 categorizes the two subdivided cleavable linker types and summarizes the main features of these linkers.

### 4.1. Cleavable Linkers

Cleavable linkers are designed to release the payload upon reaching the target cell. They have a diversity of applications but have poor stability in the blood circulation compared to non-cleavable linkers [4]. Cleavable linkers including Val-Cit, N-Succinimidyl 4-(2-pyridyldithio) butanoate (SPDB), N-succinimidyl-4-(2-pyridyldithio) pentanoate (SPP) and hydrazide are cleaved succeeding internalization. For these linkers cleavage is generally either triggered by protease reactions such as glutathione reduction or acidic pH [72,73].

Cleavable linkers tend to increase the bystander effect and are favored when designing ADCs that target antigens which are heterogeneously expressed in tumors [74,75]. Upon internalization, the linker is cleaved by a specific protease or by a defined pH and the free drug is released [75]. This free drug is designed to directly kill the antigen positive target cell, it can in some cases diffuse out of the target cell and subsequently kill the surrounding antigen negative cells causing a bystander effect [50,75].

Cleavable linkers are subdivided into two categories: chemically cleavable and enzyme cleavable linkers. This will be delineated in this review. 

### 4.2. Enzyme Cleavable Linkers

#### 4.2.1. Enzyme Activable Linkers

Enzyme activable linkers are cleaved by tumor associated enzymes [68]. The success of the cathepsin B-sensitive Val-Cit dipeptide led to the development of the Glu-Val-Cit linker. When compared to the traditional Val-Cit linker, in vivo results demonstrated superior activity. The glutamic acid residue further enhanced plasma stability [49,51]. According to a study by Bargh et al., 2019 [49], the antitumor activity of Glu-Val-Cit with other linkers (Val-Cit and Ser-Val-Cit) showed stability over 14 days compared to the other linkers which almost completely hydrolyzed over the 14-day period. Insertion of a Polyethylene glycol spacer (PEG spacer) between the linker and antibody structure meant that the linker structure would be exposed to enzymatic degradation; in spite of this, the Glu-Val-Cit linker demonstrated superior therapeutic activity [51].

The success of this linker structure paved the way to improve the specificity towards the enzyme of interest. Genentech improved the specificity of the linker structure towards the enzyme of interest by replacing the Val residue in the Val-Cit dipeptide with a cyclobutane-1,1-dicarboxamide moiety (cBu-Cit linker) [52,53,68]. The linker showed similar stability in the circulation; however, in comparison to the traditional Val-Cit dipeptide, it showed superior cathepsin B specificity in tumor-bearing mice [68].

#### 4.2.2. Sulfatase-Cleavable Linkers

The novel sulfatase linker releases the payload after cleavage by lysosomal sulfatase enzymes. These linkers were analyzed for their ability to release the payload while maintaining stability in the circulation. They are overexpressed in a number of cancers. Sulfatases are found in abundance in the lysosome and upon hydrolysis, the 4-alkoxybenzyl carbamate is primed for 1,6-elimination of an amine linked payload. Sulfatase linkers are stable in human plasma and stable in mouse plasma; they are hydrophilic in nature and efficiently release the payload at the target site. In a study by Bargh et al., 2020 [54] the antibody trastuzumab and the payload MMAE was employed and tested against HER2+ (BT474) and HER2- (MCF7) cells. Compared to the controls employed in this study, the aryl sulfate containing ADCs were more potent based on their in vitro assay results. To compare the efficacy of variations of linker design, an ortho-amide group was incorporated in the synthesis of this linker. However, this design showed poor cytotoxic activity against the HER2+ cells, leading to the hypothesis that the tubulin binding ability was inhibited by the presence of an adjacent anionic sulfate group. Thereby, the ortho-amide group was eliminated in the final design of the linker. Stability and release studies by Bargh et al., 2020 [54], showed that sulfatase linker linked at the benzyl position was hydrolyzed after a longer period of time and the rate of cleavage decreased with an increase in pH compared to the sulfatase linker linked at the ortho-amide position. The sulfatase-linkers can be employed for lipophilic payloads based on their solubility. They can be incorporated in the design of ADCs for the treatment of cancer.

#### 4.2.3. Galactosidase Cleavable Linker

The β-Galactosidase enzyme is over expressed in some tumors. It cleaves the β-Galactosidase cleavable linker in the lysosome through hydrolysis [56]. When this linker was employed with the antibody trastuzumab and the payload MMAE, it showed higher potency than the Valine-Citrulline para-aminobenzyloxycarbonyl (Val-Cit-PABC) analogue. Kolodych et al., 2017 [55] developed the new class of galactosidase cleavable linker for conjugation with ADCs. The in vitro cytotoxic assays compared the cytotoxic effect of the payload when attached to the galactosidase linker and the antibody trastuzumab as well as with other common cleavable and non-cleavable linkers used as controls. The galactosidase showed superior IC_50_ effects against the SKBR3 cell line. The in vivo effects also showed that the galactosidase linker is effective in delivery of the payload [55].

#### 4.2.4. Lysosomal Protease-Sensitive Linkers/Peptide-Based Linkers

Lysosomal protease-sensitive linkers/peptide-based linkers are commonly used in ADC designs. Lysosomal proteases i.e., cathepsin B are highly expressed on tumor cells [57]. Lysosomal protease linkers are stable in the systemic circulation and release the drug specifically in the target cells. The Val-Cit linker is a protease cleavable linker commonly used in the design of some ADCs such as Brentuximab vedotin [59,61]. In addition to this valine-alanine (Val-Ala), phenylalanine-lysine (Phe-Lys) has been used in some ADCs including SGN-CD70A and labetuzumab-SN-38 [58,61]. Other dipeptide linkers used in ADCs include L-Alanyl-L-alanine (Ala-Ala) [60].

#### 4.2.5. Glucuronide Linker

The β-glucuronide linker is a protease sensitive linker that is hydrolyzed by the β-glucuronidase enzyme to release the drug. Tumor necrotic regions and lysosomes are rich in β-glucuronidase which is active at lysosomal pH [63]. Successful cleavage of the glycosidic linkage enables selective cytotoxic payload release [49,59]. Hence, an ADC conjugated to a β-glucuronide linker is stable in circulation due to its selective payload release [59,64]. This linker is hydrophilic in nature. Its hydrophilic nature makes it a suitable candidate for hydrophobic payloads [64,65]. Its efficacy is comparable to ADC-Val-Cit linker based on a study that compared PEG_12_-glucuronide-MMAE and Val-Cit-MMAE [62]. 

According to Bargh et al., 2019 [49], ADC plasma exposure and activity was maximized as a result of increasing the hydrophilicity of β-glucuronic acid-based linkers with branched PEG chains, ultimately optimizing antigen mediated uptake at the tumor site.

### 4.3. Chemically Cleavable Linkers 

#### 4.3.1. Acid Sensitive Cleavable Linker

Acid sensitive cleavable linkers are designed to be stable in an alkaline environment, such as the systemic circulation, but sensitive to acidic environments [4]. They are cleaved, after internalization, into the target cell. These linkers, however, have been associated with non-specific drug release [59]. Acid cleavable linkers cannot be used to conjugate highly cytotoxic drugs [4,42,68,69].

Existing acid cleavable linkers are restricted in terms of their applicability due to their lack of stability. Attempts were made to increase the DAR of cytotoxins such as SN-38 to subsequently improve efficacy [69]. However, there is a risk of immunogenic reactions or faster clearance rates in some cases because of a high DAR [46,69]. Moreover, most of the cytotoxic agents are hydrophobic and the incorporation of many molecules can lead to decreased physical stability, the addition of branched PEG chains, and can increase the hydrophilicity of the linker, which has subsequently proven to have superior effects in vivo [47,65,66].

Furthermore, diisopropyl silyl ether-based linker is a novel acid cleavable linker, that was designed for the conjugation of monomethyl auristatin E (MMAE) to a the anti-HER2 mAb, mil40, which is a biosimilar of trastuzumab. It is a novel conjugating model for MMAE with a DAR of 5.5. The novel design by Wang et al., 2019 [69], is comprised of a silyl ether-based acid cleavable p-hydroxybenzyl alcohol (PHB) antibody-MMAE conjugate with acid triggered silyl ether groups and is said to broaden the development of antibody drug conjugates with very potent payloads. According to the study by Wang et al., 2019 [69], previously designed acid cleavable linkers including the hydrazone and carbonate linkers had a plasma half-life of 2–3 days or 1 day respectively. However, the pH sensitive silyl ether linker has a plasma half-life of >7 days. MMAE was employed since it is a potent drug that is also a commonly employed enzyme for conjugation with cleavable linkers such as the Val-Cit cathepsin B-cleavable linker. The design of this linker drug structure addresses toxicity, efficacy, stability, and efficient release of MMAE.

#### 4.3.2. Glutathione-Sensitive Disulfide Linkers

Glutathione is found in the intracellular and extracellular compartment [59]. It is a low molecular weight thiol that is released during cell stress in hypoxic conditions, cell survival and tumor growth [70,71]. Hence, a higher concentration of glutathione is found in cancer cells compared to normal cells. Glutathione sensitive linkers are cleaved as a result of an elevated concentration of glutathione in the tumor cell, subsequently releasing the payload [70]. The disulfide bond increases the stability of the linker in circulation and is cleaved upon internalization by intracellular glutathione. It has the ability to resist reductive cleavage in circulation which reduces the likelihood of off-target release/toxicity [56]. This linker had been employed for maytansinoid based drug-conjugates [4].

### 4.4. Non-Cleavable Linkers

Non-cleavable linkers remain stable from early to late endosome transition. These linkers (i.e., MCC and MC, conjugates the payload to the antibody via thioether linkage) require complete degradation of the antibody within the lysosome to release the payload. Its stability in plasma is superior to cleavable linkers [4,76,77]. Hence, cytotoxic agents are not released at off target sites, and this reduces the likelihood of harm exhibited towards healthy cells. The lack of cell permeability of non-cleavable linkers minimizes its ability to exert the bystander effect [77]. The cytotoxic drug as a result accumulates within the tumor cell and does not escape by diffusion through the cell membrane. These linkers are employed for hematological tumors and cancers with high antigen expression. An example of an ADC conjugated to a non-cleavable linker is the trastuzumab-SMCC-DM1 structure. Succinimidyl-4-[N-maleimidomethyl] cyclohexane-1-carboxylate) (SMCC) is a reducible thioether linker and DM1 is the cytotoxic payload [1,59].

**Figure 2:**
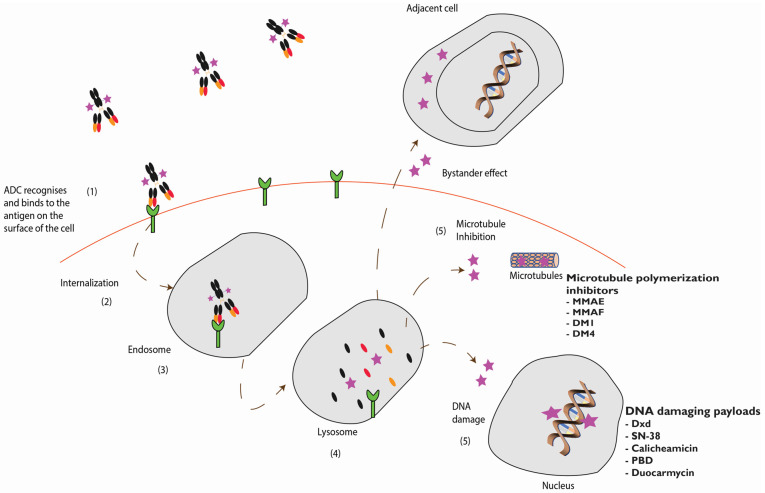
Mechanism of action for ADCs: Once the ADC binds to the antigen (green) (**1**), it is then internalized (**2**) by a process called receptor mediated endocytosis (**3**), it is then cleaved in the lysosome (**4**) and the payload is released (**5**). Depending on the class of payload (pink), it can either inhibit microtubules (**5**) or damage DNA (**5**). This free drug is designed to directly kill the antigen positive target cell, it can in some cases diffuse out of the target cell and subsequently kill the surrounding antigen negative cells causing a bystander effect.

The impact of the bystander effect and the mechanism of action of an ADC is illustrated in Figure 2.

## 5. Payload

One of the most critical components of the ADC is the payload. The payload should exert maximal cytotoxic effects at a minimal concentration [78]. Moreover, a limitation with some anticancer drugs, including etoposide, mitoxantrone and doxorubicin (DOX), is that they are impaired in hypoxic conditions (anaerobic conditions) [79]. Tumor cells develop new blood vessels and tend to adapt to environments with low oxygen levels. Hypoxia is one of the many causes of therapy resistance [80]. This may limit the use of these drugs in the design of ADCs.

There are certain features that are essential in a cytotoxic payload: high cytotoxicity, low immunogenic effect, stability during storage in preparation and in the circulation, permeability, solubility clearance, and amenability to modification. In terms of hydrophobicity, the formation of a hydrophobic metabolite post intercellular cleavage exerts better blood clearance and safety [47]. Extreme hydrophobicity of the cytotoxic agent can alter the biological properties of the antibody and can consequently cause aggregation during storage or conjugation [47].

On the other hand, hydrophilic payloads of the ADC lead to a bystander effect [81]. The bystander effect is especially beneficial for cancers that have low and heterogeneous expression of the target antigen [46]. Hydrophobic linkers conjugated to hydrophobic payloads can contribute to aggregation of the ADC [4,47]. Aggregated proteins can provoke undesirable immunogenic effects while in the blood circulation, and this should be avoided [82].

Employing hydrophilic linkers (pyrophosphate diester groups, polyethylene (PEG) or negatively charged sulfonate groups) may overcome this issue of aggregation [4,62,65,83]. An increase in the hydrophilicity of the payload, in turn, contributes to the overall safety, stability, and hydrophilicity of the ADC to non-target cells, enabling cytotoxicity by increasing the DAR and enhancing the bystander effect [84].

Current payloads target either cell proliferation or DNA synthesis [85]. Commonly used cytotoxic payloads in clinical investigation include maytansinoids (DM1 and DM4), double strand break agents (calicheamicin), auristatins (MMAE, MMAF), DNA topoisomerase I inhibitors (SN-38, exatecan), alkylators (duocarmycin, indolinobenzodiazepine dimer- IGN), cross-linkers (pyrrolobenzodiazepine dimer- PBD) [56,78,86].

### 5.1. DNA Damaging Payloads

Double strand break agents, DNA topoisomerase I inhibitors, alkylators and cross linkers are all cytotoxic agents involved in DNA damage [87].

#### 5.1.1. Double Strand Break Agents

The double strand break agent calicheamicin targets DNA synthesis. It cleaves the DNA strand by binding to the minor groove of DNA and destroys the cancer cell. The ADCs gemtozumab ozogamicin and inotuzumab ozogamicin use calicheamicin as the payload and it is attached to the antibody via the 4-(4-acetylphenoxy) butanoic acid linker (AcBut) targets CD33 and CD22, respectively [88].

#### 5.1.2. Topoisomerases

Topoisomerases are enzymes that break and ligate one or both strands of DNA. The topoisomerase I enzyme relaxes supercoiling; it cleaves one strand of DNA and then religates the strand. However, DNA topoisomerase I inhibitors block the ligation step of the cell cycle which leads to DNA single and double strand breaks and ultimately cell death [89]. These include camptothecins such as topotecan, irinotecan, and belotecan.

The anticancer drug SN-38 is an active metabolite of irinotecan. It has been employed for use in the ADC, Sacituzumab govitecan (IMMU-132) (Trodelvy^®^) approved for Triple negative breast cancer. Other camptothecins such as topotecan and irinotecan have been approved by the FDA and in 2021 the EMA has granted accelerated approval. Topotecan was approved for ovarian and lung cancers and irinotecan was approved for colorectal cancer [90].

Topoisomerase II inhibitors cleave both strands of DNA simultaneously and then religates the strands. These include anthracyclines such as doxorubicin, daunorubicin, epirubicin, idarubicin [91,92]. Doxorubicin is used in many cancer types against solid tumors. It has been proposed that doxorubicin works by two mechanisms of action: intercalating into DNA to inhibit Topoisomerase II in the chromatin structure and oxidative damage and generation of free radicals to biomolecules [93]. Compared to doxorubicin, Epirubicin has a longer half-life and higher volume of distribution (epirubicin t_½_ = 31–35 h and doxorubicin t_½_ = 1–3 h). Daunorubicin has a methyl group whereas idarubicin is missing the 4-methoxy group but is similar in structure to daunorubicin, it is more lipophilic, and it is superior in terms of its cellular uptake compared to daunorubicin [91].

#### 5.1.3. Alkylating Agents

Alkylating agents (duocarmycin, indolinobenzodiazepine dimer- IGN) form DNA adducts and adenine alkylation at the N3 position by binding to the minor grove of DNA. This leads to cell death and apoptosis due to irreversible alkylation that impacts the structural integrity of the DNA leading to DNA cleavage. Duocarmycin has been employed in anti-HER2 ADCs such as SYD983 [86]. Second generation ADC SYD985 has demonstrated effectiveness in models that have shown resistance to first generation ADCs such as T-DM1 [94]. Moreover, duocarmycin has the potential to be effective against multidrug resistant (MDR) tumor cells. They have demonstrated cytotoxicity even in cells that express the P-glycoprotein pump (P-gp) [95].

#### 5.1.4. Crosslinkers

Cross linkers, Pyrrolobenzodiazepines (PBD) exert their cytotoxic effects by covalently binding to the minor groove of the double stranded DNA and form a crosslink between the N2 position of the guanine residue and C11 position of PBD. The formation of PBD–DNA adducts blocks cell division of the cancer cell without distortion of the double helix [86]. This prevents any potential emergence of drug resistance and evades DNA damage repair response. PBD is selective as it can only bind to double stranded DNA and not single stranded DNA [96,97]. PBD has been used as a payload for the ADC, Vadastuximab talirine or SGN-CD33A being developed by Seattle Genetics to treat acute myeloid leukemia (AML) targeting CD33 [98]. However, phase III trials were halted due to patient deaths and safety concerns [99].

### 5.2. Payloads That Inhibit Tubulin Polymerization

Maytansinoids and Auristatins are tubulin polymerization inhibitors. Microtubules are vital for many cellular processes including differentiation, motility, cell division [100].

#### 5.2.1. Maytansinoids

Maytansinoids, DM1 and DM4 target microtubules and inhibit cell proliferation at the mitotic stage of the cell cycle by binding to tubulin and disrupting the assembly of microtubules [101]. ADCs that have employed the maytansinoid DM1 include Trastuzumab-MCC-DM1 (T-DM1), which targets HER2 for the treatment of metastatic breast cancer. The ADC lorvotuzumab mertansine (huN901-SPP-DM1) has shown promising results in targeting solid and liquid tumors that express CD56. The difference between the two ADCs is that T-DM1 employed a non-cleavable thioether liker whereas lorvotuzumab mertansine employed a cleavable disulfide linker [102].

#### 5.2.2. Auristatins

Auristatins, MMAE and MMAF, blocks the polymerization of tubulin; this inhibits cell division. They cause cell cycle arrest in the G2/M phase [56]. A study that investigated the effect of MMAE in vitro and in vivo demonstrated that MMAE, when attached to the Val-Cit linker, showed high cytotoxicity in vitro and low toxicity in vivo [21]. The ADC brentuximab vedotin is composed of MMAE conjugated to the antibody via a protease cleavable linker targeting CD30 in relapsed or refractory lymphomas [103]. Recently developed Polatuzumab vedotin targeting CD79b has also employed MMAE conjugated to the antibody via engineered cysteines for the treatment of relapsed or refractory diffuse large B-cell lymphoma [104].

## 6. Limitations and Challenges Associated with ADCs

ADCs are designed to be target specific missiles. However, one of the major challenges with ADCs is off target toxicities that are a result of the cytotoxic small molecules released into the blood circulation prematurely. The risk associated increases depending on the toxicity profile linked to the cytotoxic small molecules [105]. The off-target toxicity liked to mertansine (DM1) is hepatotoxicity and thrombocytopenia; MMAE is linked to peripheral neuropathy, neutropenia and the possibility of anemia; MMAF is linked to ocular toxicity [105,106,107]. In terms of the metabolic profile of ADCs, increased clearance rises from ADC hydrophobicity due to high drug loading of hydrophobic small molecules [65,108]. An in vivo study involving xenograft models compared the effect of a high drug loading of MMAE conjugated to anti-CD30 mAb with different DARs (2, 4 and 8) on ADC clearance. The results indicated that the higher the drug loading, the higher the clearance rate. In this study, the ADC with the DAR of 8 was most rapidly cleared [109].

Furthermore, in addition to increased clearance rates, ADCs are also prone to aggregation. ADC aggregation is an obstacle in initial stages in the development of an ADC. It causes structural modification which can hinder its binding ability to the antigen. Apart from this, degradation by aggregation presents a major challenge in terms of meeting the guidelines for stability testing in order to gain eligibility for registration of the prescription medicine. Guidelines have been provided for this by the regulatory agencies such as the Australian Therapeutic Goods Administration (TGA), European Medicines Agency (EMA) and FDA. This is discussed further in Section 6.3.

Moreover, linkers have an essential role in maintaining the stability of ADCs in the systemic circulation. They are classed as non-cleavable or cleavable based on their release mechanism. The bystander effect is usually exhibited by ADCs with cleavable linkers, which has the potential to impact neighboring healthy cells [15,50,75]. It was argued that the bystander effect is not a limitation; in most cases it can be beneficial especially for cancers that have low and heterogenous expression of the target antigen [46,108,110]. Some ADCs, including brentuximab vedotin comprised of the payload MMAE conjugated via a cleavable linker, exert the bystander effect [111,112]. The disulfide linkage is another example of a cleavable linker, which exhibits bystander cytotoxicity, however, the thioether linkage, which is a non-cleavable linker, does not exhibit bystander activity [46,75]. ADCs with disulfide linkage demonstrated superior cytotoxic activity than with the thioether linkage [46]. Non-cleavable linkers are unlikely to exhibit the bystander effect as the antibody needs to be completely degraded after internalization, rather than the linker, for the drug to be released. 

Interestingly, although attachment of the PEG moiety overcomes the issues associated with decreased physical stability of the ADC as a result of hydrophobicity, the presence of anti-PEG antibodies in the circulation can detect and degrade the PEG moiety following any injection consisting of PEG polymer particles. To overcome this obstacle, the use of zwitterionic polymers can substitute for PEG. It has an advantageous prolonged circulation and resists degradation by the immune system following injection [113].

Another factor that presents a challenge is drug resistance. Payload internalization and retention is majorly impacted by acquired drug resistance mechanisms such as a low level of the target antigen on the tumor cell [105].

### 6.1. Drug Resistance

Drug resistance occurs when a treatment fails or reduces in its ability to demonstrate effectiveness or when the tumor evades or acquires ways to escape. This may occur at the beginning when treatment commences or could evolve after treatment with the drug. There are many mechanisms responsible for drug resistance, some of which include: reduction in antigen levels, drug efflux pumps, default in trafficking pathways and internalization, defect in lysosomal function, alternation in signaling pathways and apoptotic dysregulation [114,115,116,117].

A decrease in antigen levels can occur following multiple cycles of exposure to the ADC. To demonstrate this, a study by Loganzo et al., 2015 [118] exposed an anti-HER2 trastuzumab–maytansinoid ADC (TM-ADC) to JIMT1-TM resistant cells (originated from a patient who failed therapy with trastuzumab), the results obtained indicated a decrease in HER2 antigen levels months after commencing treatment [113,118]. Another mechanism of resistance is drug efflux pumps; many payloads are substrates of ABC transporters. It is the most common mechanism of resistance due to over expression of drug transporters such as P-glycoprotein pump and MDR1 [116,119,120,121]. These pumps efflux the cytotoxic agent out of the cell inhibiting its ability to exert its effects in the cell. Researchers are currently focused on the use of P-gp inhibitors to overcome this issue, particularly in cells overexpressing P-gp such as Caco-2 (colorectal carcinoma cells) [119]. Additionally, as a result of impaired lysosomal function, some ADCs (e.g., T-DM1) fail to release the payload prior to the commencement of the recycling mechanism; factors such as diminished lysosomal acidification or protease exposure to resistance result in low proteolytic enzyme activity which is generally responsible for ADC degradation [115,122]. A proposed mechanism of resistance is changes to signaling pathways involving mutations of PIK3CA linked to low clinical responses for antibodies such as pertuzumab [115]. Additionally, mutations of the target antigen and single nucleotide polymorphisms (SNP) lead to resistance over time and impact the efficacy of ADC therapeutics. A study found that with the ADC, gemtuzumab ozogamicin (Mylotarg^®^: Pfizer/Wyeth), the introduction of an SNP in the splice enhancer section of CD33 resulted in a lack of the gene exon 2, and thereby reduced levels of the CD33 antigen. This genotype labelled CC, interestingly, indicated that individuals who had the CC genotype had a lower risk of relapse following treatment with gemtuzumab ozogamicin, as opposed to patients with other forms of the SNP genotypes such as CT or TT [122,123,124]. Table 3 summarizes the proposed mechanisms of drug resistance.

### 6.2. Strategies to Overcome Resistance

#### 6.2.1. Functionalizing Auristatins to Overcome MDR

Compared to MMAE, the use of monomethyl auristatin F (MMAF) analogs though functionalization of the N-terminal of the N-methylvaline of MMAF can reduce membrane permeability and bystander killing. A recent study by Moquist et al., 2020 [125], has shown that N-terminus alkyl MMAF derivatives increases hydrophobicity but, more importantly, membrane permeability and the potential to overcome MDR. 

To assess the benefit of these modifications Moquist et al., 2020 [125] assessed the N- alkylated MMAF analogs against HL60 (acute promyelocytic leukemia). This cell line expresses low levels of P-glycoprotein and has the MDR phenotype. It was also tested against the HL60/RV cell line which over expresses P-glycoprotein [126]. Overall, it showed that alkyl chain length had an impact on the IC_50_ values; a chain length of C5-C6 alkyl groups has the most optimal outcome. Chain length beyond C6 had a higher IC_50_ possibly related to an increase on susceptibility to the efflux pumps [125].

#### 6.2.2. Linker Modification

Linker modification can overcome issues, particularly with MDR1 expressing tumors. The use of a more hydrophilic linker is another strategy to overcome MDR since the transporter MDR1 transports hydrophobic compounds more effectively than hydrophilic compounds. The addition of the PEG moiety demonstrated an increase in potency in some studies, for example, the addition of the hydrophilic PEG_4_Mal linker to the MCC-linker of T-DM1 demonstrated improved potency in vitro and in vivo in MDR1- expressing tumor models [115,121]. Hydrophilic linkers such as sulfo-SPDB and mal-PEG4-N-hydroxysuccinimide have shown increased potency in MDR1 positive models. A study that incorporated NCI-N87 gastric carcinoma cancer cells to generate T-DM1 resistant cells by exposing the cells to multiple dosing cycles with T-DM1, has also shown that by replacing the non-cleavable linker with a protease cleavable linker, MC-Val-Cit-PAB also known as Valine-Citrulline monomethyl auristatin E (VcMMAE), can effectively overcome acquired T-DM1 resistance. The cells were highly sensitive to the linker modification [118,127].

#### 6.2.3. Integrating ADCs with Other Targeted Agents and Immune Checkpoint Inhibitors (ICI)

When combining ADCs with other cytotoxic agents, the two drugs should not have overlapping adverse effect profiles to avoid additive toxicities [122]. To prevent cross resistance, the combined cytotoxic agents should have unique modes of action. A phase Ib/IIa metastatic breast cancer trial tested combining T-DM1 with docetaxel. Docetaxel plus T-DM1 was effective, however, approximately half of the patients experienced severe adverse effects that required dose reductions [128]. The use of ADCs targeting the same antigen entails the risk of tumor cells evolving different mechanisms of escape that exploit aspects shared by both ADCs. There are multiple ongoing clinical trials for ADCs in combination with small molecule inhibitors and standard chemotherapeutics including clinical trials for HER2+ metastatic breast cancer, NCT03429101 (T-DM1 and poziotinib targeting Covalent HER1/2/4 kinase inhibitor) in phase I and NCT02657343 (T-DM1 and ribociclib targeting CDK4/6 inhibitor) in phase Ib/II [11,122].

Immune checkpoint inhibition (ICI) enhances antitumor immune responses of effector T-cells while stimulating antitumor immunological memory in patients [129]. ICI antibody therapy only works well for tumors that have already an ongoing antitumor T-cell response. It allows the immune system to destroy tumor cells regardless of whether the antigen has downregulated or completely lost the surface antigen that the ADC targets, this amplifies its benefit as a combination agent [122]. There are a number of ADCs that have combined immune checkpoint inhibitor agents in clinical trials, some examples include T-DM1 and atezolizumab (NCT02924883) in phase Ib for the treatment of locally advanced or metastatic HER2+ breast cancer. Its mechanism of action involves targeting programmed cell Death-Ligand 1 (PD-L1) blocking antibody [11]. Brentuximab vedotin and nivolumab (NCT02581631) in phase I/II for the treatment of relapsed/refractory CD30+ Hodgkin Lymphoma [11]. Its mechanism of action also involves targeting PD-L1. PD-L1 is a protein highly expressed on tumor cells. It is normally responsible for inhibiting T-cell activation and tends to protect tumors from T-cells in cancer. Apart from PD-L1, another ICI of interest is cytotoxic T-lymphocyte associated antigen 4 (CTLA4) [129,130,131,132].

### 6.3. Protein Aggregation

Protein aggregation is a major hurdle for the development of ADCs. It can occur at every stage of development as well as during transport and long-term storage. Aggregates can be immunogenic too. Further, protein aggregation leads to product loss, and overall any chemical or physical degradation could lead to structural modification of the ADC and cause excessive protein aggregation. There are also various other factors that can lead to aggregation such as frequent freeze/thaw cycles, high protein and salt concentrations, elevated temperature or low pH. In addition, most payloads are hydrophobic and conjugating payloads at a high DAR on the surface of the protein causes excessive protein aggregation which hinders successful development of ADCs. In some ADCs, payloads are conjugated to Cys residues after breaking existing disulfide bonds. This causes a lot of issues including aggregation, as structural integrity of the protein is lost and aggregation prone regions (hydrophobic regions that are generally buried inside protein) are exposed [9,10], and hydrophobic–hydrophobic interactions are increased. 

In general, managing protein aggregation is challenging since each mAb has varied stability requirements based on the variation between complementarity determining region (CDR) of different mAbs and this is due to the amino acids that are exposed on the surface of the mAb that are responsible for antigen specificity [2,9]. However, there are characterization techniques, such as dye binding assays and size exclusion chromatography-high performance liquid chromatography (SEC-HPLC), for early detection of presence of aggregates during design and development. SEC-HPLC is a commonly used characterization technique to obtain the aggregation profile of a mAb. Nevertheless, it is not cost effective, and can be tedious and laborious [133,134]. Dye binding experiments can be advantageous in analyzing the stability of the mAb preceding conjugation. They are fluorescent based experiments that are not restricted by protein quantity. The actual dye binding assays require minimal amounts of protein and are fairly fast paced experiments. A study that investigated the presence of aggregation and protein unfolding using two different dyes, thioflavin (ThT) and the hydrophobic dye 1-anilino-8-naphthale-neosulfonate (ANS) respectively. In this study, an mAb was incubated at 150 mg/mL at temperatures ranging from 63–70 °C. The results indicated that only bound ThT emitted a high level of fluorescence (i.e., ThT bound to aggregates) compared to unbound ThT. The fluorescence emission of the ANS dye indicated protein unfolding [133]. Using these testing methods can be useful to evidently demonstrate that the biological product (i.e., protein) can pass aggregation tests along with other analytical tests to determine the stability of the product, is vital step for product registration.

Furthermore, there are many potential anomalies (i.e., aggregation/degradation) that can arise during the manufacture process, long term storage and shipping, as mentioned above. The TGA, EU and FDA have strict guidelines for stability testing preceding the registration and transport of a prescription medicine [135]. Since temperature fluctuations can play a pivotal role in aggregation, the guidelines recommend the provision of real time data to justify the acceptable period of time that the drug product can remain out of the refrigerator before being returned to its recommended temperature. Characterization assays, such as enzyme linked immunosorbent assay (ELISA), and high-performance liquid chromatography (HPLC) are not deemed sufficient, since it does not account for the presence of impurities and potential changes to the protein structure before, during and after alterations in the manufacturing process. Temperature cycling studies are also recommended to account for the variation in temperature, particularly for cold chain products during shipping, and to demonstrate that the product will maintain its stability [135].

## 7. Concluding Remarks and Outlook

This review provides an insight into recent advances in the development of ADCs for the treatment of cancer and summarizes approved products. Furthermore, it outlines the integral features of current and newly developed linkers. Linkers fall into one of two categories: cleavable or non-cleavable. They have an important role in maintaining the stability of the ADC to avoid premature release of the highly cytotoxic drug cargo in the circulation which leads to an array of systemic toxicities. The cytotoxic drug cargo has a pivotal role in exerting cell death and apoptosis upon reaching the tumor site. They are classed depending on their ability to damage DNA or inhibit tubulin polymerization. The features and application of the payloads used in ADC conjugation are delineated in this review.

Amidst the success and excitement in the progress of ADC development lies other complexities and limitations, including rapid ADC clearance and drug resistance mechanisms that hinder these target specific missiles from exerting their optimal effects. Outlined in this review are some proposed strategies to overcome these limitations. Protein aggregation is one of the major hurdles in ADC development; management and control of aggregation can be challenging for ADCs. The review illustrates the common causes of aggregation and characterization techniques that can assist in detecting the presence of aggregates. Further investigation regarding the pharmacokinetics and pharmacodynamics profiles of the proposed strategies as well as other mechanisms of resistance that tumor cells use to evade treatment is required. 

## Figures and Tables

**Table 1:** Approved ADCs for clinical use.

Brand Name	Other Names	Manufacturer	Indication	Linker	Payload	Target	DAR, Half-Life, Clearance	Approval Year US	Approval Year EU	Reference
Adcetris^®^	Brentuximab vedotin SGN-35 cAC10-vcMMAE	Seattle genetics	Hematological malignancies relapsed or refractory HL and ALCL	Val-Cit	MMAE	CD30	DAR: 4 Half-life: 4–6 days Clearance: 1.457 L/day	2011	Conditional approval in 2012	[19,27]
Besponsa^®^	Inotuzumab ozogamicin CMC-544	Pfizer /Wyeth	Hematological malignancies relapsed or refractory ALL	AcBut	Calicheamicin	CD22	DAR: 6 Half-life: 12.3 days Clearance: 0.0333 L/day	2017	2017	[19,30]
Kadcyla^®^	rastuzumab emtansine	Genentech /Roche	Metastatic breast cancer	SMCC	DM1	HER2	DAR: 3.5 Half-life: 4 days Clearance: 0.68 L/day	2013	2013	[31]
Mylotarg^®^	Gemtuzumab ozogamicin	Pfizer /Wyeth	Acute myeloid leukemia	AcBut	Calicheamicin	CD33	DAR: average 2–3 Half-life: 62 h after the first dose and 90 h after the second dose Clearance: 0.35 L/hour	Approved in 2000 initially and reapproved in 2017	2018	[24]
Polivy^®^	Polatuzumab vedotin	Genentech /Roche	Relapsed or refractory diffuse large B-cell lymphoma	Val-Cit	MMAE	CD79b	DAR: 3.5 Half-life: 12 days Clearance: 0.9 L/day	2019	2020	[19,33]
Trodelvy^®^	Sacituzumab govitecan (IMMU-132)	Gilead Sciences/ Immunomedics Inc	Triple negative breast cancer	hydrolysable linker CL2A	SN-38	TROP-2	DAR: 7.6 Half-life: 16 h Clearance: 0.002 L/hour/kg	2020	2021 accelerated approval	[19,36]
Enhertu^®^	[fam]-trastuzumab deruxtecan	Daiichi Sankyo/ AstraZeneca	HER2+ metastatic breast cancer	clea- vable tetrapeptide based linker	DXd	HER2	DAR: 8 Half-life 5.7–5.8 days Clearance: 0.42 L/day	Late 2019	2021 conditional approval	[19,37]
Padcev^®^	Enfortumab vedotin	Astellas Pharma /Seattle Genetics	Urothelial cancer	Val-Cit	MMAE	Nectin 4	DAR: 3.8 Half-life: 3.4 days Clearance: 0.10 L/hour	Late 2019	NA	[19,40]
Blenrep^®^	Belantamab mafodotin	Glaxo -SmithKline	Multiple myeloma	MC	MMAF	BMCA	DAR: 4 Half-life: 12 days after the first dose and 14 days at steady state Clearance: 0.9 L/day	2020	2020	[19,41]

**Table 2:** Enzyme and chemically cleavable linkers.

Linker	Linker Type	Features	Reference
Enzyme activable linker	Enzyme cleavable	Glu-Val-Cit cleaved by cathepsin B stable in the circulation for over 14 days addition of the glutamic acid residue further enhances plasma stability in vivo results indicated superior cathepsin B specificity with the glutamic acid residue cBu-Cit linker cleaved by cathepsin B improved the specificity of the linker structure towards the enzyme of interest in vivo results indicated superior cathepsin B specificity after replacing the val residue in Val-Cit moiety with cyclobutane-1,1-dicarboxamide (cBu)	[25,49,51,52,53]
Sulfatase linker	Enzyme cleavable	cleaved by lysosomal sulfatase enzymes ability to release the payload while maintaining stability in the circulation hydrophilic in nature suitable candidate to employ hydrophobic payloads stable in human and mouse plasma	[54]
β-galactosidase cleavable linker	Enzyme cleavable	cleaved in the lysosome through hydrolysis by the β-Galactosidase enzymes has higher potency than the Val-Cit-PABC analogue effectively delivers the payload demonstrated superior in vitro and in vivo activity compared to other cleavable and non-cleavable linkers	[55,56]
Lysosomal protease-sensitive linkers/peptide-based linkers	Enzyme cleavable	cleaved by cathepsin B stable in the systemic circulation commonly used in the design of some ADCs, such as Brentuximab vedotin other protease cleavable linker variations include being investigated include ala-ala, phe-lys and val-ala	[57,58,59,60,61]
β-glucuronide linker	Enzyme cleavable	hydrolyzed by β-glucuronidase stable in circulation due to its selective payload release tumor necrotic regions and lysosomes are rich in β-glucuronidase hydrophilic in nature hydrophilicity and uptake into the tumor cells is increased with the addition of branched PEG chains suitable candidate for hydrophobic payloads	[49,59,62,63,64,65]
Acid sensitive linker	Chemically cleavable	AcBut linker stable in an alkaline environment, such as the systemic circulation, but sensitive to acidic environments tends to be associated with non-specific drug release used in the design of some ADCs: Inotuzumab ozogamicin and Gemtuzumab ozogamicin diisopropyl silyl ether-based linker novel acid cleavable linker plasma half-life of >7 days suitable for conjugation with highly potent payloads efficient payload release at the target site	[4,42,46,59,65,66,67,68,69]
Glutathione-sensitive disulfide linkers	Chemically cleavable	cleaved by glutathione glutathione is found in the intracellular and extracellular compartment higher concentration of glutathione is found in cancer cells compared to normal cells hydrophilic nature makes it a suitable candidate for lipophilic payloads ability to resist reductive cleavage in circulation which reduces the likelihood of off-target release/toxicity	[4,56,59,70,71]

**Table 3:** Proposed mechanisms of drug resistance.

Mechanism of Resistance	Cause of Resistance	Reference
Decrease in antigen levels	Multiple cycles of exposure to the ADC resulting in: decrease in antigen expression on the tumor cell Blanketed binding site or potential mutation Eradicated binding site	[113,118]
Drug efflux pumps	Over expression of the P-glycoprotein pump or MDR1 drug transporter	[116,119,120,121]
Defect in lysosomal function	Drug reaches the lysosomal compartment but accumulates in the lysosome due to: low acid levels in the lysosome which affects the cleavage of acid sensitive linkers decrease in proteolytic activity in the lysosome	[115,116,117]
Alternation in signalling pathways	Caused by changes and manipulation in signalling pathways tends to enhance survival and activate of anti-apoptotic factors. Some of the pathways that are manipulated and lead to apoptotic resistance are listed below: PI3K/AKT JAK/STAT Bcl-2/Bcl-xl	[115,116,122]

## Data Availability

Not applicable.

## Abbreviations

AcBut	4-(4-acetylphenoxy) butanoic acid
ADC	Antibody drug conjugates
ALCL	anaplastic large cell lymphoma
ALL	Acute lymphoblastic leukaemia
AML	acute myeloid leukaemia
ANS	1-anilino-8-naphthale-neosulfonate
BCMA	B cell maturation antigen
cBu	cyclobutane-1,1-dicarboxamide
CDR	Complementarity determining region
CL2A	cleavable PEG8- and triazole-containing PABC–peptide–MC linker
CTLA4	cytotoxic T-lymphocyte associated antigen 4
Cys	Cysteine
DAR	Drug-antibody-ratio
DM1	derivative of maytansine/mertansine
DNA	Deoxyribonucleic Acid
DOX	doxorubicin
DXd	deruxtecan
ELISA	enzyme linked immunosorbent assay
EMA	European medicines agency
ER	estrogen receptor
EU	European Union
FDA	Food and drug administration
HER2	human epidermal growth factor receptor 2
HL	Hodgkin lymphoma
HPLC	High performance liquid chromatography
ICI	Immune checkpoint inhibition
IgG	Immunoglobulin G
IV	intravenous
Lys	lysine
mAb	Monoclonal antibody
MC	maleimidocaproyl
MCC	maleimidomethyl cyclohexane-1-carboxylate
MC-Val-Cit	Maleimidocaproyl Valine-Citrulline
MDR	Multi drug resistance
MMAE	Monomethyl auristatin E
MMAF	Monomethyl auristatin F
P-gp	P-glycoprotein
PAB	para-aminobenzyl
PBD	Pyrrolobenzodiazepines
PD-L1	Programmed cell Death-Ligand 1
PEG	Polyethylene glycol
PHB	p-hydroxybenzyl alcohol
PR	progesterone receptor
SC	subcutaneous
SEC-HPLC	size exclusion chromatography-high performance liquid chromatography
SMCC	Succinimidyl-4-[N-maleimidomethyl] cyclohexane-1-carboxylate)
SN-38	active metabolite of the topoisomerase I inhibitor irinotecan
SNP	single nucleotide polymorphisms
SPDB	N-succinimidyl-4-(2-pyridyldithio) butanoate
SPP	N-succinimidyl-4-(2-pyridyldithio) pentanoate
T-DM1	Trastuzumab emtansine
TGA	Therapeutic goods administration
ThT	thioflavin
TROP-2	tumor associated calcium signal transducer 2
Val-CitPABC	Valine-Citrulline para-aminobenzyloxycarbonyl
Val-Cit	Valine-Citrulline
VcMMAE	Valine-Citrulline monomethyl auristatin E
